# Predictors of Defensive Practices among Italian Psychiatrists: Additional Findings from a National Survey

**DOI:** 10.3390/medicina59111928

**Published:** 2023-10-31

**Authors:** Donato Morena, Nicola Di Fazio, Pasquale Scognamiglio, Giuseppe Delogu, Benedetta Baldari, Luigi Cipolloni, Paola Frati, Vittorio Fineschi

**Affiliations:** 1 Department of Anatomical, Histological, Forensic and Orthopaedic Sciences, Sapienza University of Rome, 00185 Rome, Italy; donato.morena@uniroma1.it (D.M.); nicola.difazio@uniroma1.it (N.D.F.); giuseppe.delogu@uniroma1.it (G.D.); benedetta.baldari@uniroma1.it (B.B.); vittorio.fineschi@uniroma1.it (V.F.); 2 Department of Mental Health, ASL Napoli 3 Sud, 80059 Torre del Greco, Italy; 3 Department of Clinical and Experimental Medicine, University of Foggia, 71100 Foggia, Italy; luigi.cipolloni@unifg.it

**Keywords:** defensive medicine, professional liability, malpractice, position of guarantee, duty of care, forensic psychiatry, violence, survey, guidelines, evidence-based medicine

## Abstract

*Background*: Defensive medicine is characterized by medical decisions made primarily as a precaution against potential malpractice claims. For psychiatrists, professional responsibility encompasses not only the appropriateness of diagnosis and treatment but also the effects of their interventions on patients and their behaviors. *Objective*: To investigate the socio-demographic, educational, and occupational characteristics and work-related attitudes that may serve as predictors of defensive medicine among Italian psychiatrists. This research extends the results of a previous analysis based on a national survey. *Methods*: A secondary analysis of the database of a national survey on attitudes and behaviors of Italian psychiatrists regarding defensive medicine and professional liability was performed for this study. *Results*: Among 254 surveyed psychiatrists, 153 admitted to practicing defensive medicine, while 101 had this attitude with less than half of their patients. The first group was predominantly comprised of women (*p* = 0.014), who were younger in age (43.34 y 9.89 vs. 48.81 y 11.66, *p* < 0.001) and had fewer years of professional experience (12.09 y ± 9.8 vs. 17.46 y ± 11.2, *p* < 0.001). There were no significant differences in prior involvement in complaints (*p* = 0.876) or the usual place of work (*p* = 0.818). The most prominent predictors for practicing defensive medicine were (1) considering guidelines and good clinical practices not only for their clinical efficacy but also or exclusively for reducing the risk of legal complaints for professional liability (OR = 3.62; 95%CI, 1.75–7.49), and (2) hospitalizing patients with violent intentions even if not warranted according to their mental state (OR = 2.28; 95%CI, 1.50–3.46, *p* < 0.001). Prioritizing protection from professional liability over patients’ actual needs in prescribing or adjusting drug dosages and in involuntary hospitalization, as well as prescribing lower dosages than recommended for pregnant patients, were identified as additional predictors. Finally, years of professional experience exhibited a protective function against defensive practices. *Conclusions*: Psychiatrists advocate the need to implement a ‘risk management culture’ and the provision of more balanced duties in order to ensure ethical and evidence-based care to their patients. A particular source of concern stems from their professional responsibility towards not only the health of patients but also their behavior. However, these aspects conflict with a limited potential for assessment and intervention based on effective clinical tools. A reform of professional liability that considers the specificities of patients cared for by mental health services could contribute to reducing the risk of defensive medicine.

## 1. Introduction

Defensive medicine (DM) refers to professional practices primarily implemented to reduce exposure to malpractice liability [1]. It encompasses prescribing unnecessary or repetitive tests, procedures, and referrals (‘positive’ DM) and avoiding caring for high-risk patients or performing risky procedures (‘negative’ DM) [2]. DM has a significant impact on healthcare costs, estimated to range from 2.4 to 10% of the U.S. medical expenditure [3].

Psychiatry is considered to be among the low-risk specialties [4], with less than 1% of all medical malpractice claims paid [5]. However, despite the low incidence, there are concerns regarding the elevated likelihood of disciplinary actions compared to other specialties [6] and of criminal lawsuits in cases of patient suicide or involvement in violent actions [7].

Among physicians, defensive practices have been associated with various factors, including the legal environment, limited clinical experience, perceived susceptibility to lawsuits, and prior medico-legal issues [8]. Italian psychiatrists constitute a critical group for study due to their unique legal context. In the 1970s, Italy underwent mental health reforms, leading to a shift towards primarily outpatient, community-based care [9]. This resulted in the establishment of territorial facilities where psychiatrists care for patients, even those with serious disorders and complex rehabilitative needs. However, a reduction in personnel and resources for local services [10], along with regulatory developments that mandated the closure of forensic psychiatric hospitals [11], has created a particularly challenging working environment.

A further stressor arises from the fact that Italian physicians are legally held responsible for the outcomes of their interventions on patients and their behaviors in accordance with the legal principle known as the ‘position of guarantee’ (PoG). This specific duty encompasses a set of protective obligations that doctors have toward their patients, including their responsibility to neutralize all potential sources of harm (as outlined in Article 40 of the Italian Criminal Code). In the case of psychiatrists, this liability extends to the prevention of patients from engaging in self-harm or exhibiting harmful behaviors towards others [12,13].

A recent medical liability reform, Law 24/2017, also known as ‘Gelli-Bianco’, has aimed to improve healthcare safety by emphasizing the reinforcement of risk management and adherence to clinical guidelines. Nevertheless, our recent survey indicates that the results are still not widely perceived as successful [14].

Among 254 physicians surveyed, nearly two-thirds admitted to practicing DM and expressed concerns about liability laws, perceiving that their duty of care compromised the healthcare goals [14]. Approximately one-fifth had been involved in a complaint, reporting symptoms such as anxiety, anger, restlessness, loss of energy, and functional impairment.

The issues related to professional liability thus contribute to an increase in stress and burnout levels, which are already high for psychiatrists and often linked to factors that are beyond clinical care, such as handling situations involving violence or behavioral disorders [15].

Managing liability risks by identifying factors that predict attitudes towards DM practice could therefore have an effect on both improving care standards by informing policies to promote patient-centered and evidence-based care and the occupational quality and job satisfaction of psychiatrists.

To the best of our knowledge, no previous studies have investigated the predictors of DM in the psychiatric field, while research has focused on the predictors of DM among the other medical or surgical specialties.

Asher et al. [2] conducted a national survey in Israel with 877 physicians of different clinical (not including psychiatry) and surgical specialties, finding that independent predictors for practicing DM were a surgical specialty, a previous exposure to a lawsuit, and not performing a fellowship abroad.

Panella et al. [8], with a comprehensive study that involved 55 Italian hospitals and 1313 doctors, identified the following as predictors of DM: experience of being a second victim after an adverse event, age, years of experience, volume of activity, and specialty risk.

The aim of this report was to examine the differences among Italian psychiatrists who participated in our recent survey on DM in order to identify the variables that distinguished the group that practiced DM from those who did not. Additionally, using these variables, we sought to identify predictors associated with DM practices, with the ultimate goal of implementing priority interventions to address this phenomenon more effectively.

## 2. Materials and Methods

The study used data from a cross-sectional survey involving 254 Italian psychiatrists performed between March and April 2023 to explore their attitudes and behaviors regarding DM and professional liability [14]. A link to an anonymous questionnaire, edited and distributed using the free online tool “Google Forms” (Google LLC, Mountain View, CA, USA), was disseminated through the main means of communication in all the Italian national territory in order to obtain a homogeneous representation of professionals.

The questionnaire was based on instruments previously validated in similar studies [16,17]. The investigated domains included questions about treating suicidal (1), violent (2), pregnant (3), and elderly patients (4); initiating or changing drug treatment (5); the impact of PoG on the therapeutic relationship and decisions regarding involuntary hospitalization (6); and clinical risk management training and the relevant Italian legislation (7), specifically the so-called ‘Gelli-Bianco’ Law.

This last section consisted of multiple-choice questions, whereas the items related to the other six dimensions were scored on a Likert scale ranging from 1 (with no patient) to 5 (with every patient) (e.g., ‘Do you advise psychiatric hospitalization to a patient reporting violent intentions even if not warranted according to his/her mental state?’).

Additionally, the survey included demographic data (gender, age, education/qualification, seniority, usual place of employment, and department position) as well as questions about personal experiences related to malpractice claims, disciplinary investigations, and exposure to medico-legal literature on DM.

Regarding the age of respondents, 45 (17.7%) were under the age of 35, 135 (53.1%) were between 36 and 50 years old, and 73 (28.7%) were over 50. Approximately 60% of the sample was composed of women, with the majority being certified psychiatrists (90.9%).

Over half of the interviewees (n. 132, 52.0%) reported exposure to the risk of medical negligence that could have led to a malpractice claim, while 50 (19.7%) affirmed to have effectively received a complaint. Among these physicians, anxiety, anger, and restlessness were common reactions to legal complaints, with 40% reporting impaired functioning. The least represented emotion, disclosed by fewer than one-fifth of them, was guilt.

A stratification was performed based on the usual workplace, specifically identifying an ‘open’ group (involving those working in community mental health centers, addiction service units, and private practices—i.e., where patients and other people could access voluntarily and for a brief period) and a ‘closed’ group (involving those working in hospital wards, residential and rehabilitation centers, and mental health service units in prison—i.e., where inpatients are entrusted to healthcare personnel, in some cases even involuntarily). The ‘open’ group consisted of 175 psychiatrists (68.9%) and the ‘closed’ group of 78 (30.7%). The differences between the respondents in these two groups were investigated using a t-test for independent samples, while those between the respondents stratified by age group (‘<35’, ‘36–50’, and ‘>50’) were examined through a One-Way Analysis of Variance (ANOVA). An analysis of the correlation between acknowledgment of defensive practice and items about defensive behaviors, age, seniority, and involvement in complaints was then performed.

The main findings concerning DM were that most psychiatrists reported practicing it (n. 153/254, 60.2%) and felt that their PoG compromised their work in healthcare for patients (n. 138/253. 54.3%).

Age correlated inversely with acknowledgment of defensive practices (r = −0.245, *p* < 0.001), and younger physicians were more prone to DM, particularly for patients at risk of suicide or violence. Psychiatrists in ‘closed’ settings reported more malpractice claims (*p* = 0.037) and complaints (*p* = 0.031), as well as a greater propensity to act defensively.

In the treatment of patients with violent behavior, suicidal ideation, dual diagnoses, and criminal convictions, defensive practices were associated more with perceived legal risks (r = 0.306, *p* < 0.001) than actual legal involvement (*p* > 0.05).

Full details of the survey results are provided elsewhere [14].

## 3. Statistical Analyses

First, we performed a univariate analysis to evaluate the characteristics of physicians who engaged in DM and those who did not. The two groups were identified using the method proposed by Reuveni et al. [16]. Acknowledgment of practicing DM was based on the direct question: ‘Do you practice defensive medicine?’. Admitting to practicing DM with at least half of the patients was considered a valid cut-off. Student’s t-test for independent samples was used to analyze the differences between these groups in demographic, work, and defensive practice variables. Levene’s test was performed to verify whether the groups had significantly different variances.

Variables that emerged as significantly different in the between-group comparisons were used as covariates in a stepwise binary logistic regression analysis to explore their ability to predict DM practice.

Once the significant predictors were found, another bootstrapped logistic regression using forced entry of the predictors was performed. This analysis allows for the prediction of a dichotomous dependent variable (in this case, practice or not of DM) to be expressed in terms of odds ratio, with a bootstrap confidence interval for the Exp(B)-values, or the likelihood of a participant falling into an outcome category in response to unit changes in categorical and/or continuous independent variable(s) [18].

In the present study, participants with acknowledgment of practicing DM were coded as ‘2’, while those not practicing DM were coded as ‘1’.

Multicollinearity between individual predictors was assessed [19] and a variance inflation factor (VIF) of 4.0 was considered a tolerance value for the biasing effect of collinearity. The number of potential predictors included was less than one in every ten participants, as recommended for logistic regressions according to conservative estimates [20,21], *p*-values < 0.05 were considered to be statistically significant.

In the present study, the survey data were further processed using IBM SPSS V.25.0 statistical software.

## 4. Results

### 4.1. Between Groups Comparison of Physicians with and without Acknowledgment of Defensive Practices

According to the method proposed by Reuveni et al. [16] to discriminate on the level of DM practice, 101 psychiatrists claimed to practice DM with less than half of their patients (‘No-DM group’), while 153 admitted this attitude (‘DM-group’). The ‘DM-group’ predominantly consisted of female physicians (*p* = 0.014), who were younger (43.34 y ± 9.89 vs. 48.81 y ± 11.66, *p* < 0.001) and had fewer years of seniority (12.09 y ± 9.8 vs. 17.46 y ± 11.2, *p* < 0.001). There were no significant differences in past involvement in complaints (*p* = 0.876) or in the usual place of work (*p* = 0.818).

Regarding the questionnaire administered, the groups differed in education on medical liability, with the ‘DM-group’ reporting more frequently reading on medical-legal issues and keeping up to date on the scope of medical liability (CriminalComplaints3, *p* = 0.048). Education on this subject also had a relevant influence on their decision-making in daily clinical practice (CriminalComplaints4, *p* = 0.009).

As shown in Table 1, in the management of suicidal individuals, the ‘DM group’ tended to be more prone to defensive practices in all areas investigated. They reported more frequently resorting to unwarranted hospitalization (SuicidalPatients1, *p* = 0.001); increasing the number of follow-up visits (SuicidalPatients2, *p* = 0.001); contacting family or other support networks (SuicidalPatients3, *p* = 0.012); consulting with an experienced psychiatrist (SuicidalPatients4, *p* < 0.001); referring to another professional (SuicidalPatients5, *p* = 0.001); prescribing medication without indication (SuicidalPatients6, *p* < 0.001).

For individuals at risk of violence, the ‘DM group’ had the same attitude reported with suicidal patients, namely a greater frequency of unwarranted hospitalization (ViolentPatients1, *p* < 0.001); increased number of follow-up visits (ViolentPatients2, *p* = 0.003); consultation with an experienced psychiatrist (ViolentPatients4, *p* < 0.001); reference to another professional (ViolentlPatients5, *p* = 0.003); prescription of medication without indication (ViolentPatients6, *p* < 0.001).

The only exception was initiating contact with the family or other support networks, which both groups did with most patients (SuicidalPatients3, *p* = 0.012).

There were no differences in medication management and in the treatment of pregnant or elderly patients, except for the tendency to prescribe smaller dosages than customary (Pregnant3, *p* < 0.001; Elderly2, *p* = 0.002).

As expected, the ‘DM group’ had significantly higher values in all dimensions related to defensive practices.

Specifically, they considered the PoG to have a negative influence on clinical relationships (DefensiveMedicine2, *p* < 0.001) and outcomes (DefensiveMedicine3, *p* < 0.001) with some patients, e.g., those with violent behavior, suicidal ideation, dual diagnoses, and criminal convictions.

They also tended to prioritize the PoG over patients’ needs in prescribing drugs (DefensiveMedicine4, *p* < 0.001) and in cases of involuntary hospitalization (DefensiveMed-icine5, *p* < 0.001). Additionally, they were more inclined to hospitalize patients due to external pressures rather than as a medical necessity (DefensiveMedicine6, *p* < 0.001).

Finally, concerning risk management, the ‘DM group’ placed greater emphasis on adhering to clinical guidelines and good clinical practices as a means of safeguarding against professional liability claims (GelliBianco9, *p* < 0.001).

### 4.2. Predictors of Defensive Practices

Binary logistic regression analyses revealed that the most prominent predictors for practicing DM were 1) considering guidelines and good clinical practices not only for their clinical efficacy but also or exclusively for reducing the risk of losing in the event of complaints for professional liability (GelliBianco9, OR = 3.62; 95%CI, 1.75–7.49, *p* = 0.001); and 2) hospitalizing patients with violent intentions even if not warranted according to their mental state (ViolentPatients1, OR = 2.28; 95%CI, 1.50–3.46, *p* < 0.001).

Other predictors included prioritizing the PoG over patients’ needs in prescribing or changing the dosage of drugs (DefensiveMedicine4, OR = 1.65; 95%CI, 1.03–2.63. *p* = 0.036) and in involuntary hospitalization (DefensiveMedicine5, OR = 1.98; 95%CI, 1.08–3.60 *p* = 0.026), as well as prescribing a smaller dosage than customary for pregnant patients (Pregnant3, OR = 1.40; 95%CI, 1.07–1.81 *p* = 0.012). Finally, years of professional experience exhibited a protective function against DM (OR = 0.95; 95%CI, 0.92–0.98, *p* = 0.001). No significant collinearity emerged between individual predictors. The results of the regression analysis are reported in Table 2.

## 5. Discussion

To our knowledge, this is the first study examining potential predictors of DM in psychiatry. Unlike previous research, we opted not to employ prearranged variables to explore possible predictors of DM. Instead, we included those that were found to be significantly different between physicians practicing DM and those who do not. Before discussing the identified predictors, it is important to note the absence of secondary victim status and prior experience with professional liability among them. In this regard, several studies have presented conflicting findings regarding the impact of physicians’ liability experiences and past involvement in complaints on defensive behaviors [2,22,23], despite recent recognition of them as the main predictors of DM [8].

Specifically, in our study, there were no differences regarding past involvement in complaints or cases of negligence, nor in internal investigations in the service or in summonses to a departmental disciplinary committee.

These results are similar to those of other surveys among high-risk specialist physicians working in a volatile malpractice environment and with a substantial burden of concern about professional liability [1].

This could be explained by the fact that, although at low risk of reporting professional liability involvement, psychiatrists are burdened by strong stress linked to other factors [15], such as dealing with suicidal patients [24], those who are violent [25], or those exhibiting abnormal behaviors due to substance intoxication [26]. Furthermore, they often have a duty of care for people with personality disorders (including antisocial) [9], for which there are few therapeutic means at their disposal.

The high stress suffered by psychiatrists in their daily practice is also reflected in the feelings that have emerged among those who have been involved in a past civil or criminal complaint by one of their patients or their family members [14]. Anxiety, anger, and restlessness were common reactions, along with impaired functioning, while the least represented emotion, reported by less than one-fifth of the sample, was guilt.

This last result differs from that of other studies, where high levels of feelings of shame and guilt were found among physicians involved in litigation [3].

As suggested in our previous study on this issue [14], this could be due to various determinants and in particular to the belief that patients’ conditions are beyond physicians’ control. Specifically, this last point represents a notable and specific stress trigger for the psychiatric specialty compared to other branches of medicine.

The discrepancy between clinical practice and medico-legal risk is witnessed by the fact that the most prominent predictor for practicing DM among psychiatrists surveyed was their adherence to guidelines and good clinical practices not only for their effectiveness but also, or exclusively, for reducing the risk of sentence in case of litigation.

Other results suggest that psychiatrists who prioritize reducing legal liability over providing the best clinical care for patients may be more at risk of DM.

In this sense, hospitalizing violent patients, even if it is not warranted by their clinical condition, provides strong evidence of medical self-protection against hypothetical accusations.

It is worth noting that people with psychiatric disorders, particularly schizophrenia spectrum, personality, and substance use disorders, that is, the majority of patients who come to the attention of public psychiatric or addiction services, have an increased relative risk of acting violently [27]. The implementation of defensive measures by psychiatrists is influenced by the PoG they have with respect to these patients, together with the lack of tools that allow the risk of violence to be confidently estimated, given the heterogeneity of the possible causes [27].

Excessive attention to one’s own professional liability can also lead to prioritizing unnecessary medical interventions for violent people who mask their conduct with malingered psychiatric symptoms (mostly psychotic symptoms) in order to avoid legal repercussions for their conduct [28].

The concerns related to their duties are highlighted by further predictors of DM. These include factors such as prioritizing the PoG over patients’ needs in prescribing or changing the dosage of drugs and involuntary hospitalization.

In addition, another predictor, prescribing lower-than-optimal doses of medications, especially for vulnerable groups like pregnant women, represents a clear effort to minimize risks of liability claims based on side effects, complications, and possible healthcare damages. Nevertheless, several studies on this point show no clear information for modifications in pharmacological doses for antidepressants [29], antipsychotics [30], and some mood stabilizers [31].

Clear indications for the use of low dosages and precise timing methods during pregnancy exist for lithium [32], while some drugs should not be prescribed at all (e.g., valproate, carbamazepine), or only for a limited time (e.g., benzodiazepines) [33,34].

Consistent with findings in other studies [16,35,36], another notable point was that physicians with extensive professional experience were observed to be at a lower risk of engaging in DM. This could be due both to greater personal and professional skills self-developed during practice but also to progressive acquisitions with specific educational programs. In our previous study, in fact, we found a significantly heightened level of participation in clinical risk management training among senior psychiatrists, while a minimal percentage was provided by medical schools’ programs.

This data is alarming considering that clinical risk management, audit, and medical debriefing sessions can contribute to the improvement of defensive practices, as suggested by several studies [37,38] and by the same physicians [2].

Finally, although attitudes toward suicidal patients had not been identified as predictors, some were significantly more frequent among psychiatrists who admitted to practicing DM.

In some cases, these behaviors could be considered virtuous, such as in the case of contacts with family members or consultations with senior psychiatrists. However, some practices were not based on scientific evidence, including hospitalizations or unnecessary drug prescriptions [39].

Some behaviors can be potentially ambiguous, such as the increase in the number of follow-up visits, which may be driven more by personal medical protection than clinical necessity. The same applies to referring to another professional (for example, a psychologist and/or a social worker), which can serve both as a way to expand the support network for patients and to dilute the duty of care or potential responsibilities in case of claims.

For violent patients, almost the same attitudes were observed.

Significant inter-group differences also emerged regarding physicians’ gender, but this variable was neither a predictor nor significant in the correlation test performed in our previous study. Therefore, it could be assumed that there are no substantial gender differences with regard to DM.

Collectively, these findings suggest that DM may be driven by apprehensions regarding PoG, insufficient knowledge of guidelines (as indicated by not evidence-based use of medications during pregnancy), and limited professional experience. Concerns about professional liability determine clinical behaviors oriented towards self-protection, as demonstrated by the application of the guidelines mainly for both clinical and legal purposes.

It should be considered that these results might have been influenced by recent Italian legal precedents which are progressively holding psychiatrists accountable for violent acts committed by their patients, encompassing not only self-harm and suicide but also harm towards others [13]. However, the alignment with international data on DM indicates that these issues are universally prevalent [16].

## 6. Limits

As reported by Panella et al. [8], an important limit in studies investigating DM is the lack of its rigorous assessment and the elusiveness of its determinants.

Further, the use of an ad hoc survey for collecting self-report data is subject to social desirability and recall biases [40]. Another critical point is given by the lack of data on psychiatrists who did not accept the invitation to participate in the survey.

Regarding the so-called ‘socially desirable response bias’, which could lead to an underestimation of the true prevalence of DM due to reluctance to admit such practice [8,41], our study avoided an opposite risk, i.e., emotional responses following some criminal events involving Italian psychiatrists that occurred after the survey closure [42].

Questions on informed consent and its assessment were not included, despite representing a fundamental factor in the patient’s self-determination [43,44,45].

Finally, a larger sample would have allowed the detection of smaller effects and provided more statistical power for assessing relationships between variables.

## 7. Conclusions

In summary, this study reveals concerning patterns of defensive practices among Italian psychiatrists driven by perceived legal vulnerabilities rather than patient needs. The results underscore the urgency of implementing professional responsibility reforms and interventions to promote ethical and evidence-based healthcare. First, legislative, and institutional changes should aim to establish a better balance between professional liability and clinical autonomy.

A comprehensive approach that considers the unique duty of care challenges faced by psychiatrists should be contemplated. Second, health systems must foster a ‘risk management culture’, prioritizing patient-centered care while adequately protecting clinicians. Evidence-based risk assessment tools and shared decision making should be promoted. Third, enhanced education and supervision focused on medico-legal issues and managing uncertainty is critical, particularly for less experienced psychiatrists. Reducing knowledge gaps may help counteract anxiety fueling defensive actions.

Finally, quality improvement initiatives should incorporate metrics to monitor patient outcomes and defensive practices. A multi-pronged strategy across individual, organizational, and policy levels is required to protect both patient welfare and psychiatrist wellbeing.

This study represents an initial step in elucidating predictors of defensive medicine among Italian psychiatrists. Further investigation is essential to guide effective reforms and interventions. A collaborative, non-punitive approach that balances clinical care with legal considerations can foster the growth of psychiatric practice in the best interest of patients.

## Figures and Tables

**Table 1:** Comparison between groups practicing and not-practicing DM.

	‘No-DM Group’ n. 101 Mean (SD)	‘DM Group’ n. 153 Mean (SD)	t	df	*p*
*Criminal Complaints*					
Being involved in a malpractice case (CriminalComplaints1)	1.51(0.502)	1.46 (0.500)	0.893	252	0.373
Being involved in an internal inquiry and/or convocated by a disciplinary board (CriminalComplaints2)	1.82 (0.385)	1.85 (0.359)	−0.589	252	0.556
**Education on medical liability (CriminalComplaints3)**	1.47 (0.501)	1.34 (0.475)	1.993	206.12	0.048
**Influence of education on medical liability in clinical practice (CriminalComplaints4)**	2.29 (0.677)	2.57 (0.734)	−2.641	189	0.009
*Suicidal Patients*					
**Advise unwarranted hospitalization (SuicidalPatients1)**	2.69 (1.147)	3.18 (1.052)	−3.457	252	0.001
**Increase follow-up (SuicidalPatients2)**	3.63 (1.120)	4.08 (0.892)	−3.350	180.52	0.001
**Initiate contact with family (SuicidalPatients3)**	3.55 (1.127)	3.90 (0.933)	−2.523	185.96	0.012
**Consult senior psychiatrist (SuicidalPatients4)**	2.58 (1.042)	3.20 (1.095)	−4.444	252	<0.001
**Refer to another professional (SuicidalPatients5)**	2.52 (1.073)	2.96 (1.044)	−3.273	250	0.001
**Prescribe medication without indication (SuicidalPatients6)**	2.30 (0.920)	2.95 (1.087)	−5.055	232.65	<0.001
*Violent Patients*					
**Advise unwarranted hospitalization (ViolentPatients1)**	2.06 (0.827)	2.61 (1.002)	−4.735	237.64	<0.001
**Increase follow-up (ViolentPatients2)**	2.88 (1.057)	3.30 (1.136)	−2.959	251	0.003
Initiate contact with family (ViolentPatients3)	3.36 (1.106)	3.54 (1.082)	−1.253	251	0.211
**Consult senior psychiatrist (ViolentPatients4)**	2.72 (1.115)	3.38 (1.026)	−4.820	252	<0.001
**Refer to another professional (ViolentPatients5)**	2.31 (1.046)	2.72 (1.053)	−3.009	248	0.003
**Prescribe medication without indication (ViolentPatients6)**	2.78 (1.079)	3.37 (1.050)	−4.294	251	<0.001
*Patients Medication*					
Inform about severe yet rare side effects (PatientsMedication1)	3.84 (1.214)	3.67 (1.267)	1.095	252	0.275
Record that explained about side effects (PatientsMedication2)	2.80 (1.497)	2.54 (1.455)	1.375	252	0.170
Inform of increased risk of suicidality (PatientsMedication3)	2.52 (1.418)	2.20 (1.313)	1.829	200.17	0.069
*Pregnant patients*					
Avoid medication altogether (Pregnant1)	2.88 (1.166)	3.03 (1.197)	−0.959	251	0.338
Collect different consent (Pregnant2)	3.24 (1.753)	3.52 (1.674)	−1.290	251	0.198
**Prescribe a smaller dosage (Pregnant3)**	3.08 (1.440)	3.72 (1.285)	−3.598	194.49	<0.001
*Elderly patients*					
Inform of cerebrovascular diseases risk (Elderly1)	3.29 (1.445)	2.97 (1.407)	1.765	251	0.079
**Prescribe a smaller dosage (Elderly2)**	4.00 (0.974)	4.35 (0.781)	−3.124	251	0.002
*Defensive Medicine*					
**Admission of practicing defensive medicine (DefensiveMedicine1)**	1.85 (0.357)	3.70 (0.726)	−26.920	235.75	<0.001
**Believe the position of guarantee influences physicians’ relationships with certain types of patients, e.g., those with violent behavior, suicidal ideation, dual diagnoses, or criminal convictions (DefensiveMedicine2)**	2.37 (1.070)	3.21 (1.128)	−5.904	251	<0.001
**Believe the position of guarantee adversely affects the clinical outcome of certain types of patients, e.g., those with violent behavior, suicidal ideation, dual diagnoses, or criminal convictions (DefensiveMedicine3)**	2.40 (1.044)	3.16 (1.220)	−5.269	233.40	<0.001
**Prioritize the position of guarantee over patient needs in prescribing drugs (DefensiveMedicine4)**	1.92 (0.787)	2.64 (1.036)	−6.267	245.3	<0.001
**Prioritize the position of guarantee over patient needs in involuntary hospitalization (DefensiveMedicine5)**	1.49 (0.595)	2.14 (0.996)	−6.467	248.63	<0.001
**Involuntarily hospitalization due to external pressure (rather than a medical need) (DefensiveMedicine6)**	1.51 (0.643)	1.93 (0.817)	−4.385	251	<0.001
*‘Gelli-Bianco’—Law 24/2017*					
Kind of legal area at greatest risk for own job (GelliBianco1)	2.50 (0.772)	2.63 (0.627)	−1.380	180.88	0.169
Education on law 24/2017- so-called Gelli–Bianco law (GelliBianco2)	1.97 (0.822)	1.98 (0.862)	−0.095	251	0.924
Opinion on increased guarantees for psychiatrists provided by Law 24/2017 (GelliBianco3)	2.51 (0.586)	2.58 (0.582)	−0.702	175	0.484
Legal area where Law 24/2017 increased guarantees for psychiatrists (GelliBianco4)	2.17 (0.852)	1.99 (0.869)	1.297	167	0.196
Participation in training on clinical risk (GelliBianco5)	1.39 (0.489)	1.41 (0.494)	−0.406	252	0.685
Adequacy of clinical risk management training (GelliBianco7)	2.22 (0.725)	2.31 (0.589)	−1.056	174.36	0.292
Opinion on the utility of risk management in reducing medical claims (GelliBianco8)	1.11 (0.311)	1.11 (0.318)	−0.136	188	0.892
**Reasons for adhering to guidelines (GelliBianco9)**	1.71 (0.520)	2.09 (0.506)	−5.792	205.52	<0.001
*Others*					
**Gender** **	1.51 (0.052)	1.67 (0.038) *	−2.489	198.6	0.014
Female, n (%)	49 (19.4)	102 (40.5)			
Male, n (%)	50 (19.8)	50 (19.8)			
Other, n (%)	1 (0.4)	0 (0)			
**Age (years)**	48.81 (11.659)	43.34 (9.895)	3.882	189.84	<0.001
**Seniority (years after specialization)**	17.46 (11.203)	12.09 (9.798)	3.811	182.29	<0.001
Involvement in complaints	1.81 (0.393)	1.80 (0.398)	0.157	252	0.876
Usual place of work ***	1.30 (0.460)	1.31 (0.376)	−0.230	251	0.818
Open group, n (%)	70 (27.7)	105 (41.5)			
Closed-group, n (%)	30 (11.9)	48 (18.9)			

* Levene’s test significant (*p* < 0.05). ** male = 1, female = 2. *** ’open group’= 1, ‘closed group’= 2. Bold values denote statistical significance at the *p* < 0.05 level.

**Table 2:** Individual predictors of DM.

Individual Predictors	B	Sign.	VIF	Exp(B)	95% C.I. for Exp(B)	
					Lower	Upper
Advise unwarranted hospitalization (for violent patients) (ViolentPatients1)	0.824	<0.001	1.121	2.280	1.502	3.461
Prioritize the position of guarantee over patient needs in prescribing drugs (DefensiveMedicine4)	0.499	0.036	1.706	1.646	1.032	2.626
Prioritize the position of guarantee over patient needs in involuntary hospitalization (DefensiveMedicine5)	0.681	0.026	1.738	1.976	1.084	3.603
Prescribe a smaller dosage (for pregnant patients) (Pregnant3)	0.333	0.012	1.042	1.396	1.075	1.813
Reasons for adhering to guidelines (GelliBianco9)	1.287	0.001	1.201	3.622	1.751	7.495
Seniority after specialization (in years)	−0.054	0.001	1.041	0.947	0.916	0.979

Model: Chi^2^ (6) = 102.193; *p* < 0.001. Cox–Snell R^2^ = 0.362; Nagelkerke R^2^ = 0.493. VIF: variance inflation factor.

## Data Availability

Not applicable.
